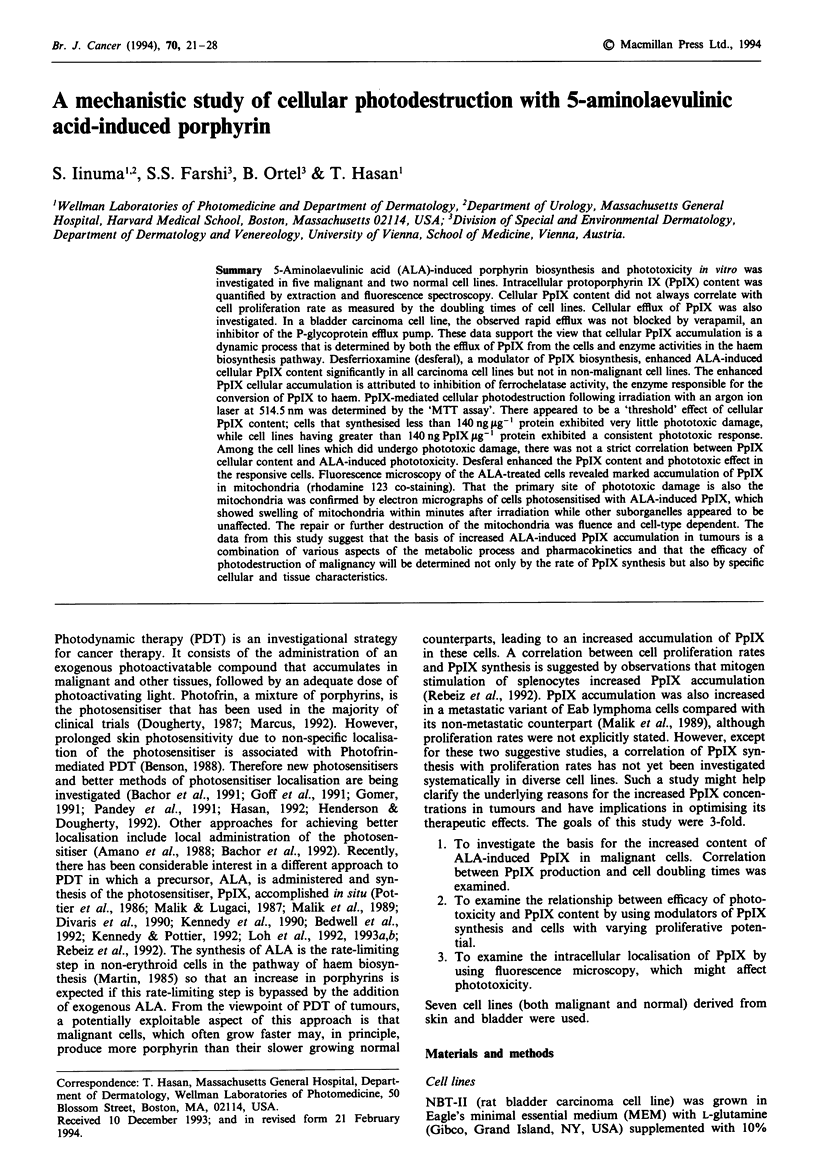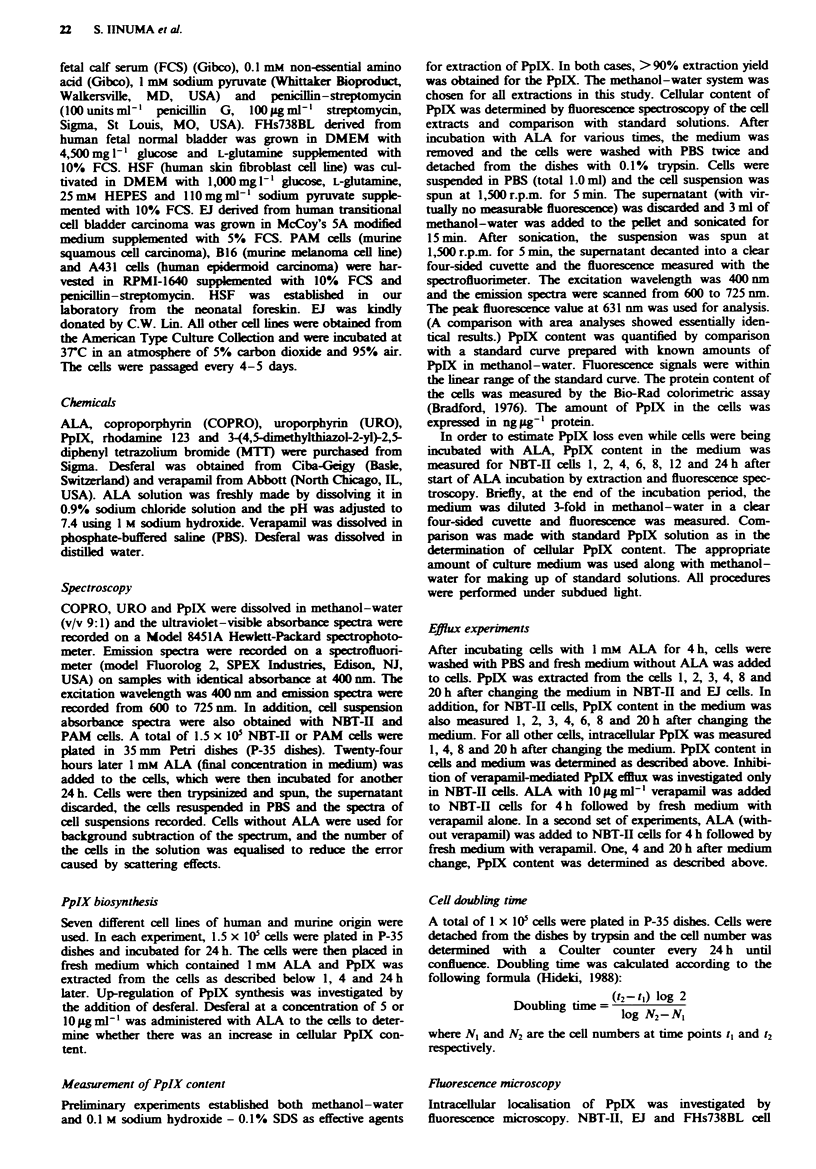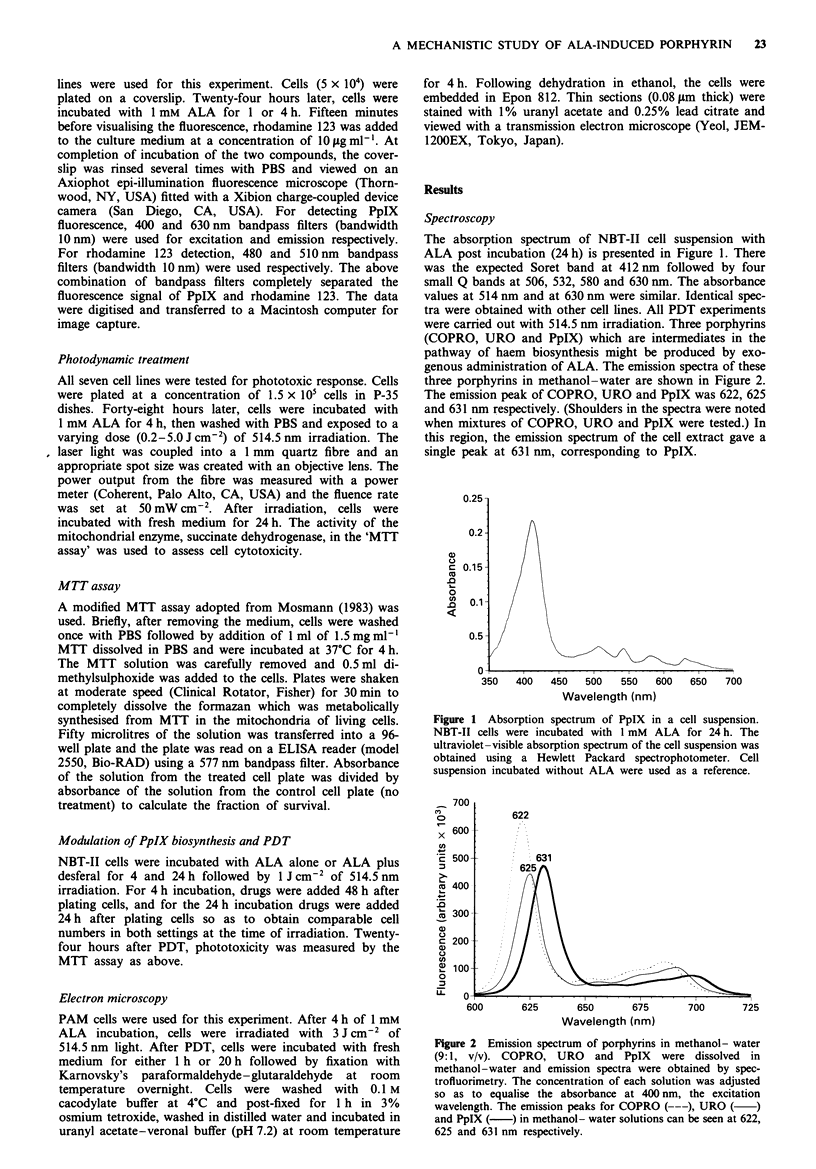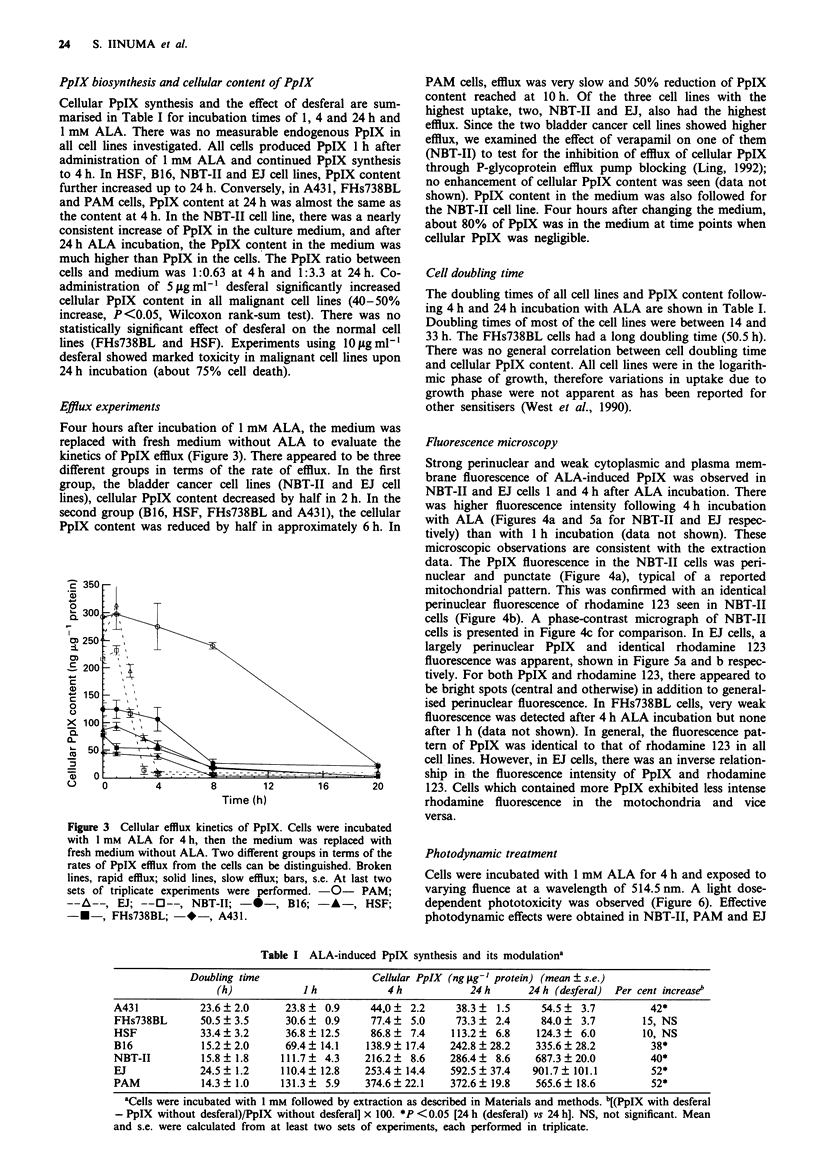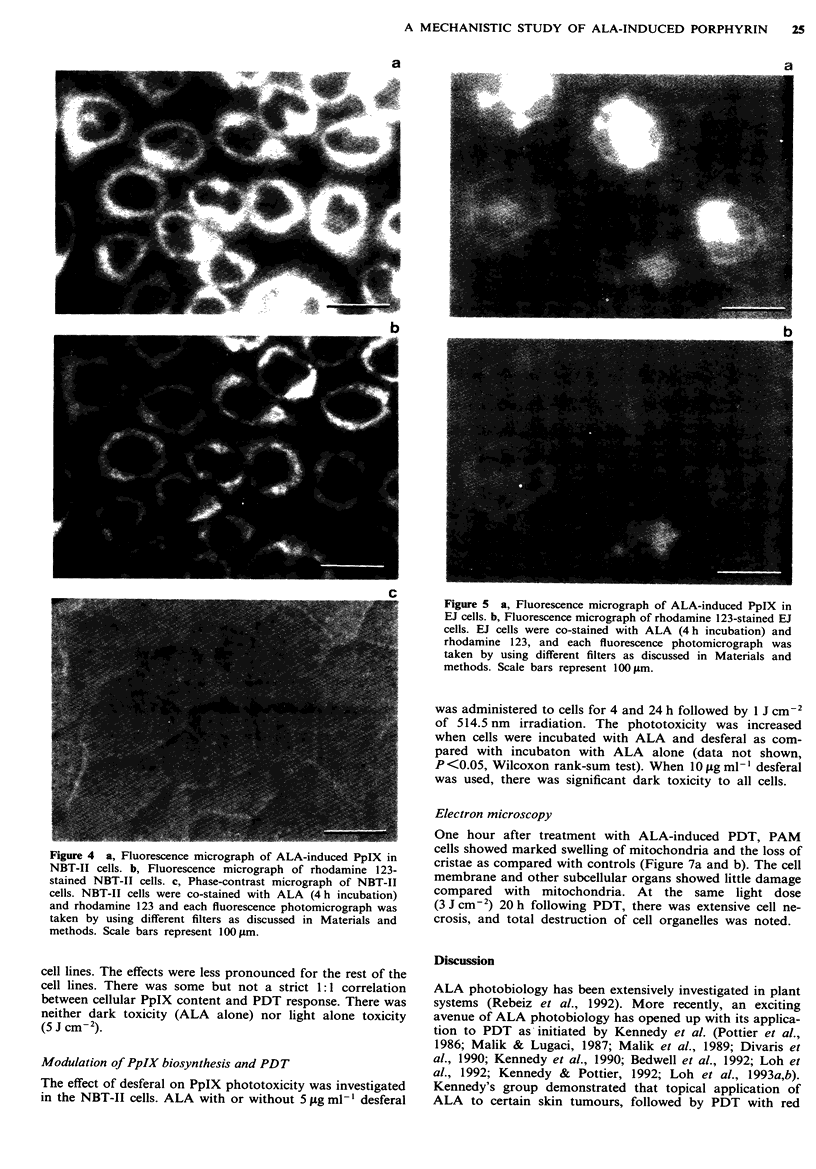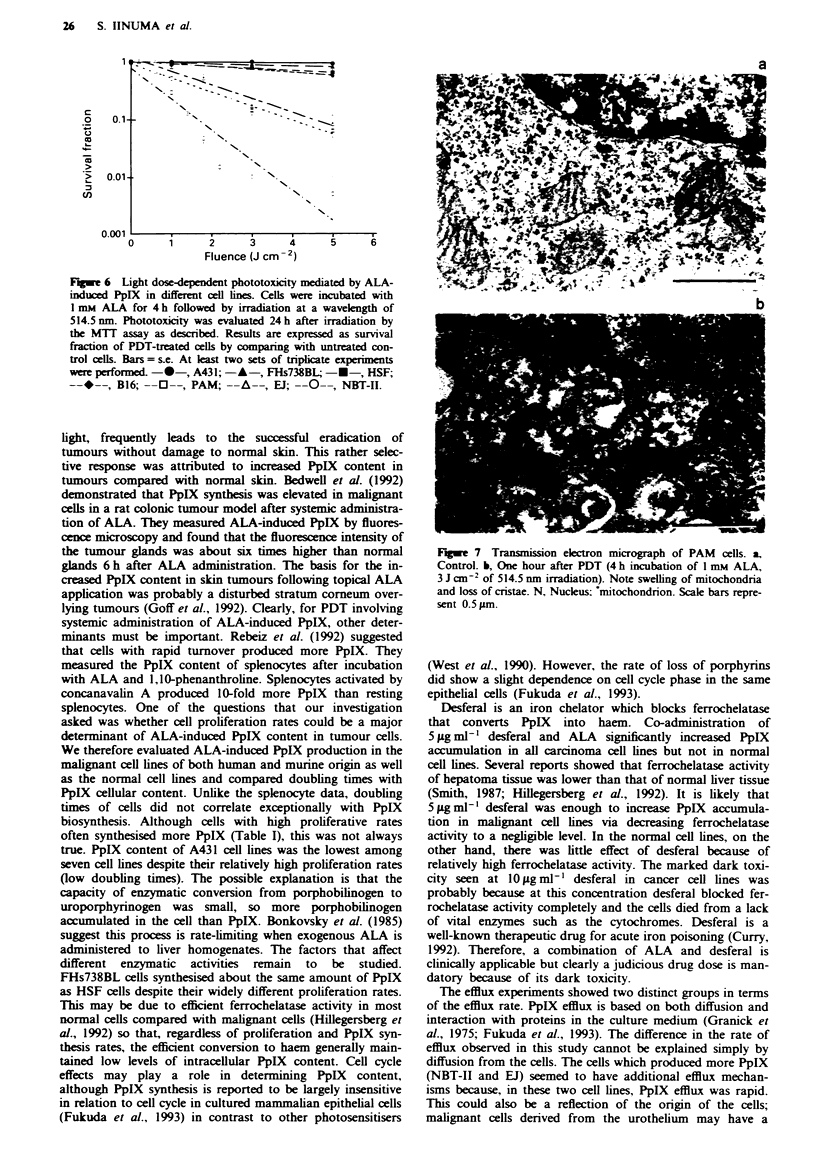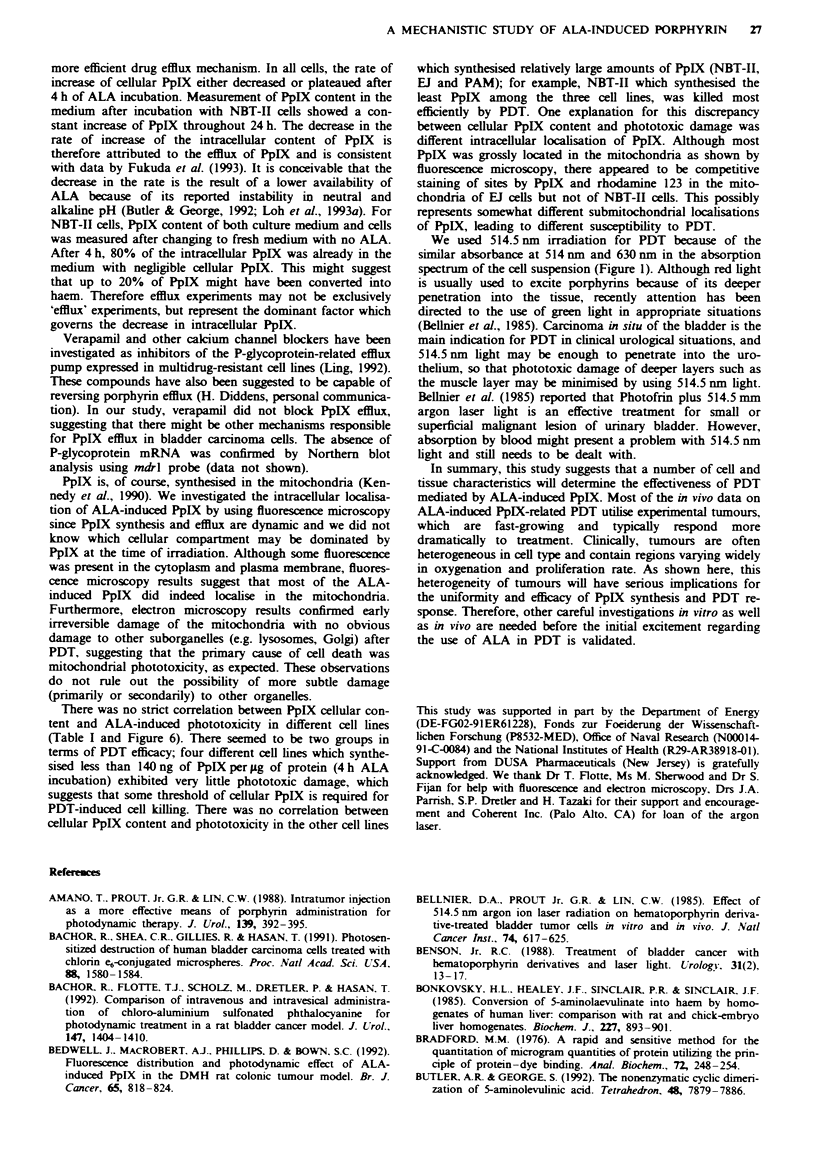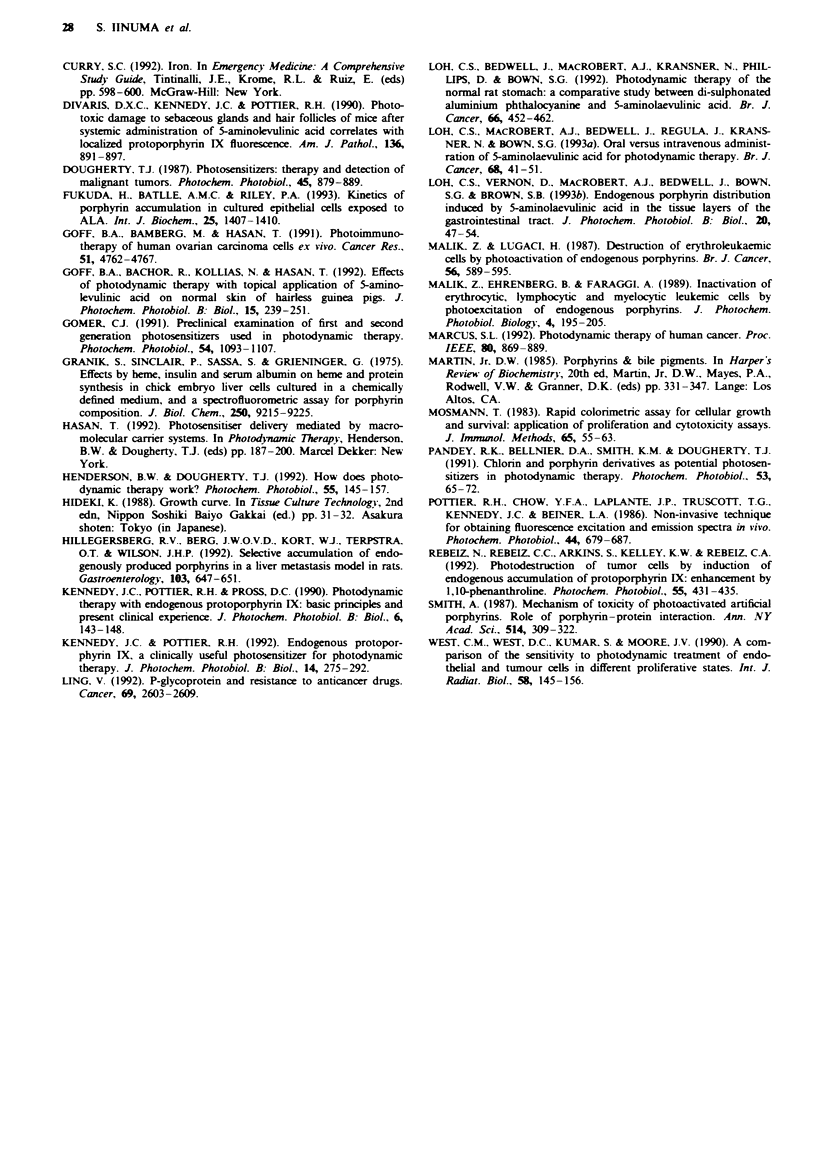# A mechanistic study of cellular photodestruction with 5-aminolaevulinic acid-induced porphyrin.

**DOI:** 10.1038/bjc.1994.244

**Published:** 1994-07

**Authors:** S. Iinuma, S. S. Farshi, B. Ortel, T. Hasan

**Affiliations:** Wellman Laboratories of Photomedicine, Massachusetts General Hospital, Boston 02114.

## Abstract

**Images:**


*Br. J. Cancer* (1994), **70**, 21–28 © Macmillan Press Ltd., 1994

### A mechanistic study of cellular photodestruction with 5-aminolaevulinic acid-induced porphyrin

S. Iinuma[1,2], S.S. Farshi[3], B. Ortel[3] & T. Hasan[1]

[1]*Wellman Laboratories of Photomedicine and Department of Dermatology,* [2]*Department of Urology, Massachusetts General Hospital, Harvard Medical School, Boston, Massachusetts 02114, USA;* [3]*Division of Special and Environmental Dermatology, Department of Dermatology and Venereology, University of Vienna, School of Medicine, Vienna, Austria.*

**Summary** 5-Aminolaevulinic acid (ALA)-induced porphyrin biosynthesis and phototoxicity *in vitro* was investigated in five malignant and two normal cell lines. Intracellular protoporphyrin IX (PpIX) content was quantified by extraction and fluorescence spectroscopy. Cellular PpIX content did not always correlate with cell proliferation rate as measured by the doubling times of cell lines. Cellular efflux of PpIX was also investigated. In a bladder carcinoma cell line, the observed rapid efflux was not blocked by verapamil, an inhibitor of the P-glycoprotein efflux pump. These data support the view that cellular PpIX accumulation is a dynamic process that is determined by both the efflux of PpIX from the cells and enzyme activities in the haem biosynthesis pathway. Desferrioxamine (desferal), a modulator of PpIX biosynthesis, enhanced ALA-induced cellular PpIX content significantly in all carcinoma cell lines but not in non-malignant cell lines. The enhanced PpIX cellular accumulation is attributed to inhibition of ferrochelatase activity, the enzyme responsible for the conversion of PpIX to haem. PpIX-mediated cellular photodestruction following irradiation with an argon ion laser at 514.5 nm was determined by the 'MTT assay'. There appeared to be a 'threshold' effect of cellular PpIX content; cells that synthesised less than 140 ng $\mu g^{-1}$ protein exhibited very little phototoxic damage, while cell lines having greater than 140 ng PpIX $\mu g^{-1}$ protein exhibited a consistent phototoxic response. Among the cell lines which did undergo phototoxic damage, there was not a strict correlation between PpIX cellular content and ALA-induced phototoxicity. Desferal enhanced the PpIX content and phototoxic effect in the responsive cells. Fluorescence microscopy of the ALA-treated cells revealed marked accumulation of PpIX in mitochondria (rhodamine 123 co-staining). That the primary site of phototoxic damage is also the mitochondria was confirmed by electron micrographs of cells photosensitised with ALA-induced PpIX, which showed swelling of mitochondria within minutes after irradiation while other suborganelles appeared to be unaffected. The repair or further destruction of the mitochondria was fluence and cell-type dependent. The data from this study suggest that the basis of increased ALA-induced PpIX accumulation in tumours is a combination of various aspects of the metabolic process and pharmacokinetics and that the efficacy of photodestruction of malignancy will be determined not only by the rate of PpIX synthesis but also by specific cellular and tissue characteristics.

Photodynamic therapy (PDT) is an investigational strategy for cancer therapy. It consists of the administration of an exogenous photoactivatable compound that accumulates in malignant and other tissues, followed by an adequate dose of photoactivating light. Photofrin, a mixture of porphyrins, is the photosensitiser that has been used in the majority of clinical trials (Dougherty, 1987; Marcus, 1992). However, prolonged skin photosensitivity due to non-specific localisation of the photosensitiser is associated with Photofrin-mediated PDT (Benson, 1988). Therefore new photosensitisers and better methods of photosensitiser localisation are being investigated (Bachor *et al.*, 1991; Goff *et al.*, 1991; Gomer, 1991; Pandey *et al.*, 1991; Hasan, 1992; Henderson & Dougherty, 1992). Other approaches for achieving better localisation include local administration of the photosensitiser (Amano *et al.*, 1988; Bachor *et al.*, 1992). Recently, there has been considerable interest in a different approach to PDT in which a precursor, ALA, is administered and synthesis of the photosensitiser, PpIX, accomplished *in situ* (Pottier *et al.*, 1986; Malik & Lugaci, 1987; Malik *et al.*, 1989; Divaris *et al.*, 1990; Kennedy *et al.*, 1990; Bedwell *et al.*, 1992; Kennedy & Pottier, 1992; Loh *et al.*, 1992, 1993*a*,*b*; Rebeiz *et al.*, 1992). The synthesis of ALA is the rate-limiting step in non-erythroid cells in the pathway of haem biosynthesis (Martin, 1985) so that an increase in porphyrins is expected if this rate-limiting step is bypassed by the addition of exogenous ALA. From the viewpoint of PDT of tumours, a potentially exploitable aspect of this approach is that malignant cells, which often grow faster may, in principle, produce more porphyrin than their slower growing normal counterparts, leading to an increased accumulation of PpIX in these cells. A correlation between cell proliferation rates and PpIX synthesis is suggested by observations that mitogen stimulation of splenocytes increased PpIX accumulation (Rebeiz *et al.*, 1992). PpIX accumulation was also increased in a metastatic variant of Eab lymphoma cells compared with its non-metastatic counterpart (Malik *et al.*, 1989), although proliferation rates were not explicitly stated. However, except for these two suggestive studies, a correlation of PpIX synthesis with proliferation rates has not yet been investigated systematically in diverse cell lines. Such a study might help clarify the underlying reasons for the increased PpIX concentrations in tumours and have implications in optimising its therapeutic effects. The goals of this study were 3-fold.

1. To investigate the basis for the increased content of ALA-induced PpIX in malignant cells. Correlation between PpIX production and cell doubling times was examined.
2. To examine the relationship between efficacy of phototoxicity and PpIX content by using modulators of PpIX synthesis and cells with varying proliferative potential.
3. To examine the intracellular localisation of PpIX by using fluorescence microscopy, which might affect phototoxicity.

Seven cell lines (both malignant and normal) derived from skin and bladder were used.

### Materials and methods

### *Cell lines*

NBT-II (rat bladder carcinoma cell line) was grown in Eagle's minimal essential medium (MEM) with L-glutamine (Gibco, Grand Island, NY, USA) supplemented with 10%

Correspondence: T. Hasan, Massachusetts General Hospital, Department of Dermatology, Wellman Laboratories of Photomedicine, 50 Blossom Street, Boston, MA, 02114, USA.
Received 10 December 1993; and in revised form 21 February 1994.

fetal calf serum (FCS) (Gibco), 0.1 mM non-essential amino acid (Gibco), 1 mM sodium pyruvate (Whittaker Bioproduct, Walkersville, MD, USA) and penicillin-streptomycin (100 units $ml^{-1}$ penicillin G, 100 $\mu g\ ml^{-1}$ streptomycin, Sigma, St Louis, MO, USA). FHs738BL derived from human fetal normal bladder was grown in DMEM with 4,500 $mg\ l^{-1}$ glucose and L-glutamine supplemented with 10% FCS. HSF (human skin fibroblast cell line) was cultivated in DMEM with 1,000 $mg\ l^{-1}$ glucose, L-glutamine, 25 mM HEPES and 110 $mg\ ml^{-1}$ sodium pyruvate supplemented with 10% FCS. EJ derived from human transitional cell bladder carcinoma was grown in McCoy's 5A modified medium supplemented with 5% FCS. PAM cells (murine squamous cell carcinoma), B16 (murine melanoma cell line) and A431 cells (human epidermoid carcinoma) were harvested in RPMI-1640 supplemented with 10% FCS and penicillin-streptomycin. HSF was established in our laboratory from the neonatal foreskin. EJ was kindly donated by C.W. Lin. All other cell lines were obtained from the American Type Culture Collection and were incubated at 37°C in an atmosphere of 5% carbon dioxide and 95% air. The cells were passaged every 4-5 days.

*Chemicals*

ALA, coproporphyrin (COPRO), uroporphyrin (URO), PpIX, rhodamine 123 and 3-(4,5-dimethylthiazol-2-yl)-2,5-diphenyl tetrazolium bromide (MTT) were purchased from Sigma. Desferal was obtained from Ciba-Geigy (Basle, Switzerland) and verapamil from Abbott (North Chicago, IL, USA). ALA solution was freshly made by dissolving it in 0.9% sodium chloride solution and the pH was adjusted to 7.4 using 1 M sodium hydroxide. Verapamil was dissolved in phosphate-buffered saline (PBS). Desferal was dissolved in distilled water.

*Spectroscopy*

COPRO, URO and PpIX were dissolved in methanol-water (v/v 9:1) and the ultraviolet-visible absorbance spectra were recorded on a Model 8451A Hewlett-Packard spectrophotometer. Emission spectra were recorded on a spectrofluorimeter (model Fluorolog 2, SPEX Industries, Edison, NJ, USA) on samples with identical absorbance at 400 nm. The excitation wavelength was 400 nm and emission spectra were recorded from 600 to 725 nm. In addition, cell suspension absorbance spectra were also obtained with NBT-II and PAM cells. A total of $1.5 \times 10^5$ NBT-II or PAM cells were plated in 35 mm Petri dishes (P-35 dishes). Twenty-four hours later 1 mM ALA (final concentration in medium) was added to the cells, which were then incubated for another 24 h. Cells were then trypsinized and spun, the supernatant discarded, the cells resuspended in PBS and the spectra of cell suspensions recorded. Cells without ALA were used for background subtraction of the spectrum, and the number of the cells in the solution was equalised to reduce the error caused by scattering effects.

*PpIX biosynthesis*

Seven different cell lines of human and murine origin were used. In each experiment, $1.5 \times 10^5$ cells were plated in P-35 dishes and incubated for 24 h. The cells were then placed in fresh medium which contained 1 mM ALA and PpIX was extracted from the cells as described below 1, 4 and 24 h later. Up-regulation of PpIX synthesis was investigated by the addition of desferal. Desferal at a concentration of 5 or 10 $\mu g\ ml^{-1}$ was administered with ALA to the cells to determine whether there was an increase in cellular PpIX content.

*Measurement of PpIX content*

Preliminary experiments established both methanol-water and 0.1 M sodium hydroxide - 0.1% SDS as effective agents for extraction of PpIX. In both cases, >90% extraction yield was obtained for the PpIX. The methanol-water system was chosen for all extractions in this study. Cellular content of PpIX was determined by fluorescence spectroscopy of the cell extracts and comparison with standard solutions. After incubation with ALA for various times, the medium was removed and the cells were washed with PBS twice and detached from the dishes with 0.1% trypsin. Cells were suspended in PBS (total 1.0 ml) and the cell suspension was spun at 1,500 r.p.m. for 5 min. The supernatant (with virtually no measurable fluorescence) was discarded and 3 ml of methanol-water was added to the pellet and sonicated for 15 min. After sonication, the suspension was spun at 1,500 r.p.m. for 5 min, the supernatant decanted into a clear four-sided cuvette and the fluorescence measured with the spectrofluorimeter. The excitation wavelength was 400 nm and the emission spectra were scanned from 600 to 725 nm. The peak fluorescence value at 631 nm was used for analysis. (A comparison with area analyses showed essentially identical results.) PpIX content was quantified by comparison with a standard curve prepared with known amounts of PpIX in methanol-water. Fluorescence signals were within the linear range of the standard curve. The protein content of the cells was measured by the Bio-Rad colorimetric assay (Bradford, 1976). The amount of PpIX in the cells was expressed in ng $\mu g^{-1}$ protein.

In order to estimate PpIX loss even while cells were being incubated with ALA, PpIX content in the medium was measured for NBT-II cells 1, 2, 4, 6, 8, 12 and 24 h after start of ALA incubation by extraction and fluorescence spectroscopy. Briefly, at the end of the incubation period, the medium was diluted 3-fold in methanol-water in a clear four-sided cuvette and fluorescence was measured. Comparison was made with standard PpIX solution as in the determination of cellular PpIX content. The appropriate amount of culture medium was used along with methanol-water for making up of standard solutions. All procedures were performed under subdued light.

*Efflux experiments*

After incubating cells with 1 mM ALA for 4 h, cells were washed with PBS and fresh medium without ALA was added to cells. PpIX was extracted from the cells 1, 2, 3, 4, 8 and 20 h after changing the medium in NBT-II and EJ cells. In addition, for NBT-II cells, PpIX content in the medium was also measured 1, 2, 3, 4, 6, 8 and 20 h after changing the medium. For all other cells, intracellular PpIX was measured 1, 4, 8 and 20 h after changing the medium. PpIX content in cells and medium was determined as described above. Inhibition of verapamil-mediated PpIX efflux was investigated only in NBT-II cells. ALA with 10 $\mu g\ ml^{-1}$ verapamil was added to NBT-II cells for 4 h followed by fresh medium with verapamil alone. In a second set of experiments, ALA (without verapamil) was added to NBT-II cells for 4 h followed by fresh medium with verapamil. One, 4 and 20 h after medium change, PpIX content was determined as described above.

*Cell doubling time*

A total of $1 \times 10^5$ cells were plated in P-35 dishes. Cells were detached from the dishes by trypsin and the cell number was determined with a Coulter counter every 24 h until confluence. Doubling time was calculated according to the following formula (Hideki, 1988):

$$\text{Doubling time} = \frac{(t_2 - t_1)\ \log\ 2}{\log\ N_2 - N_1}$$

where $N_1$ and $N_2$ are the cell numbers at time points $t_1$ and $t_2$ respectively.

*Fluorescence microscopy*

Intracellular localisation of PpIX was investigated by fluorescence microscopy. NBT-II, EJ and FHs738BL cell lines were used for this experiment. Cells ($5 \times 10^4$) were plated on a coverslip. Twenty-four hours later, cells were incubated with 1 mM ALA for 1 or 4 h. Fifteen minutes before visualising the fluorescence, rhodamine 123 was added to the culture medium at a concentration of $10\,\mu g\,ml^{-1}$. At completion of incubation of the two compounds, the coverslip was rinsed several times with PBS and viewed on an Axiophot epi-illumination fluorescence microscope (Thornwood, NY, USA) fitted with a Xibion charge-coupled device camera (San Diego, CA, USA). For detecting PpIX fluorescence, 400 and 630 nm bandpass filters (bandwidth 10 nm) were used for excitation and emission respectively. For rhodamine 123 detection, 480 and 510 nm bandpass filters (bandwidth 10 nm) were used respectively. The above combination of bandpass filters completely separated the fluorescence signal of PpIX and rhodamine 123. The data were digitised and transferred to a Macintosh computer for image capture.

*Photodynamic treatment*

All seven cell lines were tested for phototoxic response. Cells were plated at a concentration of $1.5 \times 10^5$ cells in P-35 dishes. Forty-eight hours later, cells were incubated with 1 mM ALA for 4 h, then washed with PBS and exposed to a varying dose ($0.2-5.0\,J\,cm^{-2}$) of 514.5 nm irradiation. The laser light was coupled into a 1 mm quartz fibre and an appropriate spot size was created with an objective lens. The power output from the fibre was measured with a power meter (Coherent, Palo Alto, CA, USA) and the fluence rate was set at $50\,mW\,cm^{-2}$. After irradiation, cells were incubated with fresh medium for 24 h. The activity of the mitochondrial enzyme, succinate dehydrogenase, in the 'MTT assay' was used to assess cell cytotoxicity.

*MTT assay*

A modified MTT assay adopted from Mosmann (1983) was used. Briefly, after removing the medium, cells were washed once with PBS followed by addition of 1 ml of $1.5\,mg\,ml^{-1}$ MTT dissolved in PBS and were incubated at 37°C for 4 h. The MTT solution was carefully removed and 0.5 ml dimethylsulphoxide was added to the cells. Plates were shaken at moderate speed (Clinical Rotator, Fisher) for 30 min to completely dissolve the formazan which was metabolically synthesised from MTT in the mitochondria of living cells. Fifty microlitres of the solution was transferred into a 96-well plate and the plate was read on a ELISA reader (model 2550, Bio-RAD) using a 577 nm bandpass filter. Absorbance of the solution from the treated cell plate was divided by absorbance of the solution from the control cell plate (no treatment) to calculate the fraction of survival.

*Modulation of PpIX biosynthesis and PDT*

NBT-II cells were incubated with ALA alone or ALA plus desferal for 4 and 24 h followed by $1\,J\,cm^{-2}$ of 514.5 nm irradiation. For 4 h incubation, drugs were added 48 h after plating cells, and for the 24 h incubation drugs were added 24 h after plating cells so as to obtain comparable cell numbers in both settings at the time of irradiation. Twenty-four hours after PDT, phototoxicity was measured by the MTT assay as above.

*Electron microscopy*

PAM cells were used for this experiment. After 4 h of 1 mM ALA incubation, cells were irradiated with $3\,J\,cm^{-2}$ of 514.5 nm light. After PDT, cells were incubated with fresh medium for either 1 h or 20 h followed by fixation with Karnovsky's paraformaldehyde–glutaraldehyde at room temperature overnight. Cells were washed with 0.1 M cacodylate buffer at 4°C and post-fixed for 1 h in 3% osmium tetroxide, washed in distilled water and incubated in uranyl acetate–veronal buffer (pH 7.2) at room temperature for 4 h. Following dehydration in ethanol, the cells were embedded in Epon 812. Thin sections (0.08 µm thick) were stained with 1% uranyl acetate and 0.25% lead citrate and viewed with a transmission electron microscope (Yeol, JEM-1200EX, Tokyo, Japan).

### Results

*Spectroscopy*

The absorption spectrum of NBT-II cell suspension with ALA post incubation (24 h) is presented in Figure 1. There was the expected Soret band at 412 nm followed by four small Q bands at 506, 532, 580 and 630 nm. The absorbance values at 514 nm and at 630 nm were similar. Identical spectra were obtained with other cell lines. All PDT experiments were carried out with 514.5 nm irradiation. Three porphyrins (COPRO, URO and PpIX) which are intermediates in the pathway of haem biosynthesis might be produced by exogenous administration of ALA. The emission spectra of these three porphyrins in methanol–water are shown in Figure 2. The emission peak of COPRO, URO and PpIX was 622, 625 and 631 nm respectively. (Shoulders in the spectra were noted when mixtures of COPRO, URO and PpIX were tested.) In this region, the emission spectrum of the cell extract gave a single peak at 631 nm, corresponding to PpIX.

**Figure 1** Absorption spectrum of PpIX in a cell suspension. NBT-II cells were incubated with 1 mM ALA for 24 h. The ultraviolet–visible absorption spectrum of the cell suspension was obtained using a Hewlett Packard spectrophotometer. Cell suspension incubated without ALA were used as a reference.

**Figure 2** Emission spectrum of porphyrins in methanol– water (9:1, v/v). COPRO, URO and PpIX were dissolved in methanol–water and emission spectra were obtained by spectrofluorimetry. The concentration of each solution was adjusted so as to equalise the absorbance at 400 nm, the excitation wavelength. The emission peaks for COPRO (---), URO (——) and PpIX (——) in methanol– water solutions can be seen at 622, 625 and 631 nm respectively.

*PpIX biosynthesis and cellular content of PpIX*

Cellular PpIX synthesis and the effect of desferal are summarised in Table I for incubation times of 1, 4 and 24 h and 1 mM ALA. There was no measurable endogenous PpIX in all cell lines investigated. All cells produced PpIX 1 h after administration of 1 mM ALA and continued PpIX synthesis to 4 h. In HSF, B16, NBT-II and EJ cell lines, PpIX content further increased up to 24 h. Conversely, in A431, FHs738BL and PAM cells, PpIX content at 24 h was almost the same as the content at 4 h. In the NBT-II cell line, there was a nearly consistent increase of PpIX in the culture medium, and after 24 h ALA incubation, the PpIX content in the medium was much higher than PpIX in the cells. The PpIX ratio between cells and medium was 1:0.63 at 4 h and 1:3.3 at 24 h. Co-administration of 5 $\mu g\,ml^{-1}$ desferal significantly increased cellular PpIX content in all malignant cell lines (40–50% increase, $P<0.05$, Wilcoxon rank-sum test). There was no statistically significant effect of desferal on the normal cell lines (FHs738BL and HSF). Experiments using 10 $\mu g\,ml^{-1}$ desferal showed marked toxicity in malignant cell lines upon 24 h incubation (about 75% cell death).

*Efflux experiments*

Four hours after incubation of 1 mM ALA, the medium was replaced with fresh medium without ALA to evaluate the kinetics of PpIX efflux (Figure 3). There appeared to be three different groups in terms of the rate of efflux. In the first group, the bladder cancer cell lines (NBT-II and EJ cell lines), cellular PpIX content decreased by half in 2 h. In the second group (B16, HSF, FHs738BL and A431), the cellular PpIX content was reduced by half in approximately 6 h. In PAM cells, efflux was very slow and 50% reduction of PpIX content reached at 10 h. Of the three cell lines with the highest uptake, two, NBT-II and EJ, also had the highest efflux. Since the two bladder cancer cell lines showed higher efflux, we examined the effect of verapamil on one of them (NBT-II) to test for the inhibition of efflux of cellular PpIX through P-glycoprotein efflux pump blocking (Ling, 1992); no enhancement of cellular PpIX content was seen (data not shown). PpIX content in the medium was also followed for the NBT-II cell line. Four hours after changing the medium, about 80% of PpIX was in the medium at time points when cellular PpIX was negligible.

**Figure 3** Cellular efflux kinetics of PpIX. Cells were incubated with 1 mM ALA for 4 h, then the medium was replaced with fresh medium without ALA. Two different groups in terms of the rates of PpIX efflux from the cells can be distinguished. Broken lines, rapid efflux; solid lines, slow efflux; bars, s.e. At last two sets of triplicate experiments were performed. —O— PAM; --Δ--, EJ; --□--, NBT-II; —●—, B16; —▲—, HSF; —■—, FHs738BL; —◆—, A431.

*Cell doubling time*

The doubling times of all cell lines and PpIX content following 4 h and 24 h incubation with ALA are shown in Table I. Doubling times of most of the cell lines were between 14 and 33 h. The FHs738BL cells had a long doubling time (50.5 h). There was no general correlation between cell doubling time and cellular PpIX content. All cell lines were in the logarithmic phase of growth, therefore variations in uptake due to growth phase were not apparent as has been reported for other sensitisers (West *et al.*, 1990).

*Fluorescence microscopy*

Strong perinuclear and weak cytoplasmic and plasma membrane fluorescence of ALA-induced PpIX was observed in NBT-II and EJ cells 1 and 4 h after ALA incubation. There was higher fluorescence intensity following 4 h incubation with ALA (Figures 4a and 5a for NBT-II and EJ respectively) than with 1 h incubation (data not shown). These microscopic observations are consistent with the extraction data. The PpIX fluorescence in the NBT-II cells was perinuclear and punctate (Figure 4a), typical of a reported mitochondrial pattern. This was confirmed with an identical perinuclear fluorescence of rhodamine 123 seen in NBT-II cells (Figure 4b). A phase-contrast micrograph of NBT-II cells is presented in Figure 4c for comparison. In EJ cells, a largely perinuclear PpIX and identical rhodamine 123 fluorescence was apparent, shown in Figure 5a and b respectively. For both PpIX and rhodamine 123, there appeared to be bright spots (central and otherwise) in addition to generalised perinuclear fluorescence. In FHs738BL cells, very weak fluorescence was detected after 4 h ALA incubation but none after 1 h (data not shown). In general, the fluorescence pattern of PpIX was identical to that of rhodamine 123 in all cell lines. However, in EJ cells, there was an inverse relationship in the fluorescence intensity of PpIX and rhodamine 123. Cells which contained more PpIX exhibited less intense rhodamine fluorescence in the motochondria and vice versa.

*Photodynamic treatment*

Cells were incubated with 1 mM ALA for 4 h and exposed to varying fluence at a wavelength of 514.5 nm. A light dose-dependent phototoxicity was observed (Figure 6). Effective photodynamic effects were obtained in NBT-II, PAM and EJ

**Table I** ALA-induced PpIX synthesis and its modulation[a]

| | Doubling time (h) | Cellular PpIX ($ng\,\mu g^{-1}$ protein) (mean ± s.e.) 1 h | 4 h | 24 h | 24 h (desferal) | Per cent increase[b] |
|---|---|---|---|---|---|---|
| A431 | 23.6 ± 2.0 | 23.8 ± 0.9 | 44.0 ± 2.2 | 38.3 ± 1.5 | 54.5 ± 3.7 | 42* |
| FHs738BL | 50.5 ± 3.5 | 30.6 ± 0.9 | 77.4 ± 5.0 | 73.3 ± 2.4 | 84.0 ± 3.7 | 15, NS |
| HSF | 33.4 ± 3.2 | 36.8 ± 12.5 | 86.8 ± 7.4 | 113.2 ± 6.8 | 124.3 ± 6.0 | 10, NS |
| B16 | 15.2 ± 2.0 | 69.4 ± 14.1 | 138.9 ± 17.4 | 242.8 ± 28.2 | 335.6 ± 28.2 | 38* |
| NBT-II | 15.8 ± 1.8 | 111.7 ± 4.3 | 216.2 ± 8.6 | 286.4 ± 8.6 | 687.3 ± 20.0 | 40* |
| EJ | 24.5 ± 1.2 | 110.4 ± 12.8 | 253.4 ± 14.4 | 592.5 ± 37.4 | 901.7 ± 101.1 | 52* |
| PAM | 14.3 ± 1.0 | 131.3 ± 5.9 | 374.6 ± 22.1 | 372.6 ± 19.8 | 565.6 ± 18.6 | 52* |

[a]Cells were incubated with 1 mM followed by extraction as described in Materials and methods. [b][(PpIX with desferal – PpIX without desferal)/PpIX without desferal] × 100. *$P<0.05$ [24 h (desferal) *vs* 24 h]. NS, not significant. Mean and s.e. were calculated from at least two sets of experiments, each performed in triplicate.

**Figure 4** **a**, Fluorescence micrograph of ALA-induced PpIX in NBT-II cells. **b**, Fluorescence micrograph of rhodamine 123-stained NBT-II cells. **c**, Phase-contrast micrograph of NBT-II cells. NBT-II cells were co-stained with ALA (4 h incubation) and rhodamine 123 and each fluorescence photomicrograph was taken by using different filters as discussed in Materials and methods. Scale bars represent 100 $\mu$m.

cell lines. The effects were less pronounced for the rest of the cell lines. There was some but not a strict 1:1 correlation between cellular PpIX content and PDT response. There was neither dark toxicity (ALA alone) nor light alone toxicity (5 J $cm^{-2}$).

*Modulation of PpIX biosynthesis and PDT*

The effect of desferal on PpIX phototoxicity was investigated in the NBT-II cells. ALA with or without 5 $\mu g\ ml^{-1}$ desferal

**Figure 5** **a**, Fluorescence micrograph of ALA-induced PpIX in EJ cells. **b**, Fluorescence micrograph of rhodamine 123-stained EJ cells. EJ cells were co-stained with ALA (4 h incubation) and rhodamine 123, and each fluorescence photomicrograph was taken by using different filters as discussed in Materials and methods. Scale bars represent 100 $\mu$m.

was administered to cells for 4 and 24 h followed by 1 J $cm^{-2}$ of 514.5 nm irradiation. The phototoxicity was increased when cells were incubated with ALA and desferal as compared with incubaton with ALA alone (data not shown, $P<0.05$, Wilcoxon rank-sum test). When 10 $\mu g\ ml^{-1}$ desferal was used, there was significant dark toxicity to all cells.

*Electron microscopy*

One hour after treatment with ALA-induced PDT, PAM cells showed marked swelling of mitochondria and the loss of cristae as compared with controls (Figure 7a and b). The cell membrane and other subcellular organs showed little damage compared with mitochondria. At the same light dose (3 J $cm^{-2}$) 20 h following PDT, there was extensive cell necrosis, and total destruction of cell organelles was noted.

### Discussion

ALA photobiology has been extensively investigated in plant systems (Rebeiz *et al.*, 1992). More recently, an exciting avenue of ALA photobiology has opened up with its application to PDT as initiated by Kennedy *et al.* (Pottier *et al.*, 1986; Malik & Lugaci, 1987; Malik *et al.*, 1989; Divaris *et al.*, 1990; Kennedy *et al.*, 1990; Bedwell *et al.*, 1992; Loh *et al.*, 1992; Kennedy & Pottier, 1992; Loh *et al.*, 1993*a*,*b*). Kennedy's group demonstrated that topical application of ALA to certain skin tumours, followed by PDT with red

**Figure 6** Light dose-dependent phototoxicity mediated by ALA-induced PpIX in different cell lines. Cells were incubated with 1 mM ALA for 4 h followed by irradiation at a wavelength of 514.5 nm. Phototoxicity was evaluated 24 h after irradiation by the MTT assay as described. Results are expressed as survival fraction of PDT-treated cells by comparing with untreated control cells. Bars = s.e. At least two sets of triplicate experiments were performed. —●—, A431; —▲—, FHs738BL; —■—, HSF; --◆--, B16; --□--, PAM; --Δ--, EJ; --O--, NBT-II.

light, frequently leads to the successful eradication of tumours without damage to normal skin. This rather selective response was attributed to increased PpIX content in tumours compared with normal skin. Bedwell et al. (1992) demonstrated that PpIX synthesis was elevated in malignant cells in a rat colonic tumour model after systemic administration of ALA. They measured ALA-induced PpIX by fluorescence microscopy and found that the fluorescence intensity of the tumour glands was about six times higher than normal glands 6 h after ALA administration. The basis for the increased PpIX content in skin tumours following topical ALA application was probably a disturbed stratum corneum overlying tumours (Goff et al., 1992). Clearly, for PDT involving systemic administration of ALA-induced PpIX, other determinants must be important. Rebeiz et al. (1992) suggested that cells with rapid turnover produced more PpIX. They measured the PpIX content of splenocytes after incubation with ALA and 1,10-phenanthroline. Splenocytes activated by concanavalin A produced 10-fold more PpIX than resting splenocytes. One of the questions that our investigation asked was whether cell proliferation rates could be a major determinant of ALA-induced PpIX content in tumour cells. We therefore evaluated ALA-induced PpIX production in the malignant cell lines of both human and murine origin as well as the normal cell lines and compared doubling times with PpIX cellular content. Unlike the splenocyte data, doubling times of cells did not correlate exceptionally with PpIX biosynthesis. Although cells with high proliferative rates often synthesised more PpIX (Table I), this was not always true. PpIX content of A431 cell lines was the lowest among seven cell lines despite their relatively high proliferation rates (low doubling times). The possible explanation is that the capacity of enzymatic conversion from porphobilinogen to uroporphyrinogen was small, so more porphobilinogen accumulated in the cell than PpIX. Bonkovsky et al. (1985) suggest this process is rate-limiting when exogenous ALA is administered to liver homogenates. The factors that affect different enzymatic activities remain to be studied. FHs738BL cells synthesised about the same amount of PpIX as HSF cells despite their widely different proliferation rates. This may be due to efficient ferrochelatase activity in most normal cells compared with malignant cells (Hillegersberg et al., 1992) so that, regardless of proliferation and PpIX synthesis rates, the efficient conversion to haem generally maintained low levels of intracellular PpIX content. Cell cycle effects may play a role in determining PpIX content, although PpIX synthesis is reported to be largely insensitive in relation to cell cycle in cultured mammalian epithelial cells (Fukuda et al., 1993) in contrast to other photosensitisers

**Figure 7** Transmission electron micrograph of PAM cells. **a**, Control. **b**, One hour after PDT (4 h incubation of 1 mM ALA, 3 J cm$^{-2}$ of 514.5 nm irradiation). Note swelling of mitochondria and loss of cristae. N, Nucleus; *mitochondrion. Scale bars represent 0.5 μm.

(West et al., 1990). However, the rate of loss of porphyrins did show a slight dependence on cell cycle phase in the same epithelial cells (Fukuda et al., 1993).

Desferal is an iron chelator which blocks ferrochelatase that converts PpIX into haem. Co-administration of 5 μg ml$^{-1}$ desferal and ALA significantly increased PpIX accumulation in all carcinoma cell lines but not in normal cell lines. Several reports showed that ferrochelatase activity of hepatoma tissue was lower than that of normal liver tissue (Smith, 1987; Hillegersberg et al., 1992). It is likely that 5 μg ml$^{-1}$ desferal was enough to increase PpIX accumulation in malignant cell lines via decreasing ferrochelatase activity to a negligible level. In the normal cell lines, on the other hand, there was little effect of desferal because of relatively high ferrochelatase activity. The marked dark toxicity seen at 10 μg ml$^{-1}$ desferal in cancer cell lines was probably because at this concentration desferal blocked ferrochelatase activity completely and the cells died from a lack of vital enzymes such as the cytochromes. Desferal is a well-known therapeutic drug for acute iron poisoning (Curry, 1992). Therefore, a combination of ALA and desferal is clinically applicable but clearly a judicious drug dose is mandatory because of its dark toxicity.

The efflux experiments showed two distinct groups in terms of the efflux rate. PpIX efflux is based on both diffusion and interaction with proteins in the culture medium (Granick et al., 1975; Fukuda et al., 1993). The difference in the rate of efflux observed in this study cannot be explained simply by diffusion from the cells. The cells which produced more PpIX (NBT-II and EJ) seemed to have additional efflux mechanisms because, in these two cell lines, PpIX efflux was rapid. This could also be a reflection of the origin of the cells; malignant cells derived from the urothelium may have a more efficient drug efflux mechanism. In all cells, the rate of increase of cellular PpIX either decreased or plateaued after 4 h of ALA incubation. Measurement of PpIX content in the medium after incubation with NBT-II cells showed a constant increase of PpIX throughout 24 h. The decrease in the rate of increase of the intracellular content of PpIX is therefore attributed to the efflux of PpIX and is consistent with data by Fukuda *et al.* (1993). It is conceivable that the decrease in the rate is the result of a lower availability of ALA because of its reported instability in neutral and alkaline pH (Butler & George, 1992; Loh *et al.*, 1993*a*). For NBT-II cells, PpIX content of both culture medium and cells was measured after changing to fresh medium with no ALA. After 4 h, 80% of the intracellular PpIX was already in the medium with negligible cellular PpIX. This might suggest that up to 20% of PpIX might have been converted into haem. Therefore efflux experiments may not be exclusively 'efflux' experiments, but represent the dominant factor which governs the decrease in intracellular PpIX.

Verapamil and other calcium channel blockers have been investigated as inhibitors of the P-glycoprotein-related efflux pump expressed in multidrug-resistant cell lines (Ling, 1992). These compounds have also been suggested to be capable of reversing porphyrin efflux (H. Diddens, personal communication). In our study, verapamil did not block PpIX efflux, suggesting that there might be other mechanisms responsible for PpIX efflux in bladder carcinoma cells. The absence of P-glycoprotein mRNA was confirmed by Northern blot analysis using *mdr*1 probe (data not shown).

PpIX is, of course, synthesised in the mitochondria (Kennedy *et al.*, 1990). We investigated the intracellular localisation of ALA-induced PpIX by using fluorescence microscopy since PpIX synthesis and efflux are dynamic and we did not know which cellular compartment may be dominated by PpIX at the time of irradiation. Although some fluorescence was present in the cytoplasm and plasma membrane, fluorescence microscopy results suggest that most of the ALA-induced PpIX did indeed localise in the mitochondria. Furthermore, electron microscopy results confirmed early irreversible damage of the mitochondria with no obvious damage to other suborganelles (e.g. lysosomes, Golgi) after PDT, suggesting that the primary cause of cell death was mitochondrial phototoxicity, as expected. These observations do not rule out the possibility of more subtle damage (primarily or secondarily) to other organelles.

There was no strict correlation between PpIX cellular content and ALA-induced phototoxicity in different cell lines (Table I and Figure 6). There seemed to be two groups in terms of PDT efficacy; four different cell lines which synthesised less than 140 ng of PpIX per μg of protein (4 h ALA incubation) exhibited very little phototoxic damage, which suggests that some threshold of cellular PpIX is required for PDT-induced cell killing. There was no correlation between cellular PpIX content and phototoxicity in the other cell lines which synthesised relatively large amounts of PpIX (NBT-II, EJ and PAM); for example, NBT-II which synthesised the least PpIX among the three cell lines, was killed most efficiently by PDT. One explanation for this discrepancy between cellular PpIX content and phototoxic damage was different intracellular localisation of PpIX. Although most PpIX was grossly located in the mitochondria as shown by fluorescence microscopy, there appeared to be competitive staining of sites by PpIX and rhodamine 123 in the mitochondria of EJ cells but not of NBT-II cells. This possibly represents somewhat different submitochondrial localisations of PpIX, leading to different susceptibility to PDT.

We used 514.5 nm irradiation for PDT because of the similar absorbance at 514 nm and 630 nm in the absorption spectrum of the cell suspension (Figure 1). Although red light is usually used to excite porphyrins because of its deeper penetration into the tissue, recently attention has been directed to the use of green light in appropriate situations (Bellnier *et al.*, 1985). Carcinoma *in situ* of the bladder is the main indication for PDT in clinical urological situations, and 514.5 nm light may be enough to penetrate into the urothelium, so that phototoxic damage of deeper layers such as the muscle layer may be minimised by using 514.5 nm light. Bellnier *et al.* (1985) reported that Photofrin plus 514.5 mm argon laser light is an effective treatment for small or superficial malignant lesion of urinary bladder. However, absorption by blood might present a problem with 514.5 nm light and still needs to be dealt with.

In summary, this study suggests that a number of cell and tissue characteristics will determine the effectiveness of PDT mediated by ALA-induced PpIX. Most of the *in vivo* data on ALA-induced PpIX-related PDT utilise experimental tumours, which are fast-growing and typically respond more dramatically to treatment. Clinically, tumours are often heterogeneous in cell type and contain regions varying widely in oxygenation and proliferation rate. As shown here, this heterogeneity of tumours will have serious implications for the uniformity and efficacy of PpIX synthesis and PDT response. Therefore, other careful investigations *in vitro* as well as *in vivo* are needed before the initial excitement regarding the use of ALA in PDT is validated.

This study was supported in part by the Department of Energy (DE-FG02-91ER61228), Fonds zur Foeiderung der Wissenschaftlichen Forschung (P8532-MED), Office of Naval Research (N00014-91-C-0084) and the National Institutes of Health (R29-AR38918-01). Support from DUSA Pharmaceuticals (New Jersey) is gratefully acknowledged. We thank Dr T. Flotte, Ms M. Sherwood and Dr S. Fijan for help with fluorescence and electron microscopy, Drs J.A. Parrish, S.P. Dretler and H. Tazaki for their support and encouragement and Coherent Inc. (Palo Alto, CA) for loan of the argon laser.

**References**

AMANO, T., PROUT, Jr, G.R. & LIN, C.W. (1988). Intratumor injection as a more effective means of porphyrin administration for photodynamic therapy. *J. Urol.*, **139**, 392–395.

BACHOR, R., SHEA, C.R., GILLIES, R. & HASAN, T. (1991). Photosensitized destruction of human bladder carcinoma cells treated with chlorin $e_6$-conjugated microspheres. *Proc. Natl Acad. Sci. USA*, **88**, 1580–1584.

BACHOR, R., FLOTTE, T.J., SCHOLZ, M., DRETLER, P. & HASAN, T. (1992). Comparison of intravenous and intravesical administration of chloro-aluminium sulfonated phthalocyanine for photodynamic treatment in a rat bladder cancer model. *J. Urol.*, **147**, 1404–1410.

BEDWELL, J., MACROBERT, A.J., PHILLIPS, D. & BOWN, S.C. (1992). Fluorescence distribution and photodynamic effect of ALA-induced PpIX in the DMH rat colonic tumour model. *Br. J. Cancer*, **65**, 818–824.

BELLNIER, D.A., PROUT Jr, G.R. & LIN, C.W. (1985). Effect of 514.5 nm argon ion laser radiation on hematoporphyrin derivative-treated bladder tumor cells *in vitro* and *in vivo*. *J. Natl Cancer Inst.*, **74**, 617–625.

BENSON, Jr, R.C. (1988). Treatment of bladder cancer with hematoporphyrin derivatives and laser light. *Urology*, **31**(2), 13–17.

BONKOVSKY, H.L., HEALEY, J.F., SINCLAIR, P.R. & SINCLAIR, J.F. (1985). Conversion of 5-aminolaevulinate into haem by homogenates of human liver: comparison with rat and chick-embryo liver homogenates. *Biochem. J.*, **227**, 893–901.

BRADFORD, M.M. (1976). A rapid and sensitive method for the quantitation of microgram quantities of protein utilizing the principle of protein–dye binding. *Anal. Biochem.*, **72**, 248–254.

BUTLER, A.R. & GEORGE, S. (1992). The nonenzymatic cyclic dimerization of 5-aminolevulinic acid. *Tetrahedron*, **48**, 7879–7886.

CURRY, S.C. (1992). Iron. In *Emergency Medicine: A Comprehensive Study Guide*, Tintinalli, J.E., Krome, R.L. & Ruiz, E. (eds) pp. 598-600. McGraw-Hill: New York.

DIVARIS, D.X.C., KENNEDY, J.C. & POTTIER, R.H. (1990). Phototoxic damage to sebaceous glands and hair follicles of mice after systemic administration of 5-aminolevulinic acid correlates with localized protoporphyrin IX fluorescence. *Am. J. Pathol.*, **136**, 891-897.

DOUGHERTY, T.J. (1987). Photosensitizers: therapy and detection of malignant tumors. *Photochem. Photobiol.*, **45**, 879-889.

FUKUDA, H., BATLLE, A.M.C. & RILEY, P.A. (1993). Kinetics of porphyrin accumulation in cultured epithelial cells exposed to ALA. *Int. J. Biochem.*, **25**, 1407-1410.

GOFF, B.A., BAMBERG, M. & HASAN, T. (1991). Photoimmunotherapy of human ovarian carcinoma cells *ex vivo*. *Cancer Res.*, **51**, 4762-4767.

GOFF, B.A., BACHOR, R., KOLLIAS, N. & HASAN, T. (1992). Effects of photodynamic therapy with topical application of 5-aminolevulinic acid on normal skin of hairless guinea pigs. *J. Photochem. Photobiol. B: Biol.*, **15**, 239-251.

GOMER, C.J. (1991). Preclinical examination of first and second generation photosensitizers used in photodynamic therapy. *Photochem. Photobiol.*, **54**, 1093-1107.

GRANIK, S., SINCLAIR, P., SASSA, S. & GRIENINGER, G. (1975). Effects by heme, insulin and serum albumin on heme and protein synthesis in chick embryo liver cells cultured in a chemically defined medium, and a spectrofluorometric assay for porphyrin composition. *J. Biol. Chem.*, **250**, 9215-9225.

HASAN, T. (1992). Photosensitiser delivery mediated by macromolecular carrier systems. In *Photodynamic Therapy*, Henderson, B.W. & Dougherty, T.J. (eds) pp. 187-200. Marcel Dekker: New York.

HENDERSON, B.W. & DOUGHERTY, T.J. (1992). How does photodynamic therapy work? *Photochem. Photobiol.*, **55**, 145-157.

HIDEKI, K. (1988). Growth curve. In *Tissue Culture Technology*, 2nd edn, Nippon Soshiki Baiyo Gakkai (ed.) pp. 31-32. Asakura shoten: Tokyo (in Japanese).

HILLEGERSBERG, R.V., BERG, J.W.O.V.D., KORT, W.J., TERPSTRA, O.T. & WILSON, J.H.P. (1992). Selective accumulation of endogenously produced porphyrins in a liver metastasis model in rats. *Gastroenterology*, **103**, 647-651.

KENNEDY, J.C., POTTIER, R.H. & PROSS, D.C. (1990). Photodynamic therapy with endogenous protoporphyrin IX: basic principles and present clinical experience. *J. Photochem. Photobiol. B: Biol.*, **6**, 143-148.

KENNEDY, J.C. & POTTIER, R.H. (1992). Endogenous protoporphyrin IX, a clinically useful photosensitizer for photodynamic therapy. *J. Photochem. Photobiol. B: Biol.*, **14**, 275-292.

LING, V. (1992). P-glycoprotein and resistance to anticancer drugs. *Cancer*, **69**, 2603-2609.

LOH, C.S., BEDWELL, J., MACROBERT, A.J., KRANSNER, N., PHILLIPS, D. & BOWN, S.G. (1992). Photodynamic therapy of the normal rat stomach: a comparative study between di-sulphonated aluminium phthalocyanine and 5-aminolaevulinic acid. *Br. J. Cancer*, **66**, 452-462.

LOH, C.S., MACROBERT, A.J., BEDWELL, J., REGULA, J., KRANSNER, N. & BOWN, S.G. (1993*a*). Oral versus intravenous administration of 5-aminolaevulinic acid for photodynamic therapy. *Br. J. Cancer*, **68**, 41-51.

LOH, C.S., VERNON, D., MACROBERT, A.J., BEDWELL, J., BOWN, S.G. & BROWN, S.B. (1993*b*). Endogenous porphyrin distribution induced by 5-aminolaevulinic acid in the tissue layers of the gastrointestinal tract. *J. Photochem. Photobiol. B: Biol.*, **20**, 47-54.

MALIK, Z. & LUGACI, H. (1987). Destruction of erythroleukaemic cells by photoactivation of endogenous porphyrins. *Br. J. Cancer*, **56**, 589-595.

MALIK, Z., EHRENBERG, B. & FARAGGI, A. (1989). Inactivation of erythrocytic, lymphocytic and myelocytic leukemic cells by photoexcitation of endogenous porphyrins. *J. Photochem. Photobiol. Biology*, **4**, 195-205.

MARCUS, S.L. (1992). Photodynamic therapy of human cancer. *Proc. IEEE*, **80**, 869-889.

MARTIN, Jr, D.W. (1985). Porphyrins & bile pigments. In *Harper's Review of Biochemistry*, 20th ed, Martin, Jr, D.W., Mayes, P.A., Rodwell, V.W. & Granner, D.K. (eds) pp. 331-347. Lange: Los Altos, CA.

MOSMANN, T. (1983). Rapid colorimetric assay for cellular growth and survival: application of proliferation and cytotoxicity assays. *J. Immunol. Methods*, **65**, 55-63.

PANDEY, R.K., BELLNIER, D.A., SMITH, K.M. & DOUGHERTY, T.J. (1991). Chlorin and porphyrin derivatives as potential photosensitizers in photodynamic therapy. *Photochem. Photobiol.*, **53**, 65-72.

POTTIER, R.H., CHOW, Y.F.A., LAPLANTE, J.P., TRUSCOTT, T.G., KENNEDY, J.C. & BEINER, L.A. (1986). Non-invasive technique for obtaining fluorescence excitation and emission spectra *in vivo*. *Photochem. Photobiol.*, **44**, 679-687.

REBEIZ, N., REBEIZ, C.C., ARKINS, S., KELLEY, K.W. & REBEIZ, C.A. (1992). Photodestruction of tumor cells by induction of endogenous accumulation of protoporphyrin IX: enhancement by 1,10-phenanthroline. *Photochem. Photobiol.*, **55**, 431-435.

SMITH, A. (1987). Mechanism of toxicity of photoactivated artificial porphyrins. Role of porphyrin-protein interaction. *Ann. NY Acad. Sci.*, **514**, 309-322.

WEST, C.M., WEST, D.C., KUMAR, S. & MOORE, J.V. (1990). A comparison of the sensitivity to photodynamic treatment of endothelial and tumour cells in different proliferative states. *Int. J. Radiat. Biol.*, **58**, 145-156.