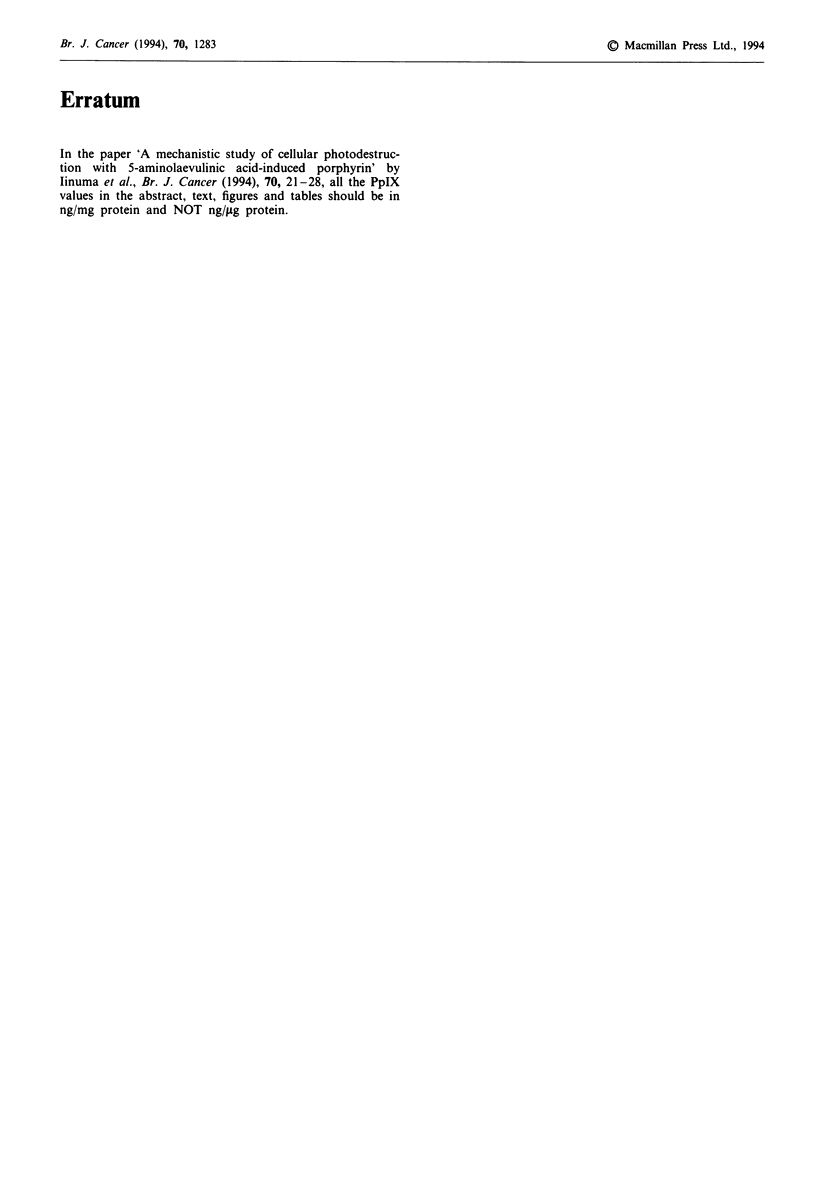# Erratum

**Published:** 1994-12

**Authors:** 


*Br. J. Cancer* (1994), **70**, 1283 © Macmillan Press Ltd., 1994

### Erratum

In the paper 'A mechanistic study of cellular photodestruction with 5-aminolaevulinic acid-induced porphyrin' by Iinuma *et al.*, *Br. J. Cancer* (1994), **70**, 21–28, all the PpIX values in the abstract, text, figures and tables should be in ng/mg protein and NOT ng/μg protein.